# Serum Transthyretin as a Prognostic and Therapeutic Biomarker in Transthyretin Amyloid Cardiomyopathy Patients Treated with Tafamidis

**DOI:** 10.3390/jcm15093355

**Published:** 2026-04-28

**Authors:** Jacopo Costantino, Federico Ballatore, Giulia Marchionni, Maria Alfarano, Silvia Stavagna, Samuel Costantino, Carmine Dario Vizza, Cristina Chimenti

**Affiliations:** 1 Department of Medical and Cardiovascular Sciences, Sapienza University of Rome, 00185 Rome, Italy; jacopo.costantino@uniroma1.it (J.C.);; 2 Department of Medicine, University of Padova, 35122 Padova, Italy

**Keywords:** transthyretin amyloid cardiomyopathy, serum transthyretin, tafamidis, prognosis, biomarkers

## Abstract

**Background:** The clinical significance of circulating serum transthyretin (sTTR) levels in patients with transthyretin amyloid cardiomyopathy (ATTR-CM) remains incompletely defined, particularly in patients treated with tafamidis in real-world clinical practice. We aimed to evaluate the relationship between sTTR levels, disease severity, longitudinal changes during tafamidis therapy, and clinical outcomes. **Methods:** We conducted a retrospective, exploratory study based on prospectively collected data, including consecutive patients with ATTR-CM initiating tafamidis therapy at a tertiary referral center. sTTR levels were measured at baseline and after six months. Associations between sTTR, markers of disease severity, functional status, and outcomes were assessed. Cox regression, receiver operating characteristic (ROC) analysis, and Kaplan–Meier survival analysis were performed. **Results:** A total of 107 patients (mean age 79.6 ± 8.5 years, 84 males) were enrolled and followed for 24 ± 6 months. Tafamidis therapy was associated with a significant increase in sTTR levels at six months (from 24.7 ± 8.8 to 36.9 ± 6.4 mg/dL; *p* = 0.006). Baseline sTTR levels were inversely correlated with NT-proBNP (ρ = –0.349; *p* = 0.037) and were significantly lower in non-survivors compared with survivors (14.2 ± 3.1 vs. 23.5 ± 5.2 mg/dL; *p* < 0.001). Each 1 mg/dL increase in baseline sTTR was associated with a 13% reduction in all-cause mortality risk (HR 0.87, 95% CI 0.83–0.91; *p* < 0.001). A baseline sTTR cut-off of 20 mg/dL was associated with an increased risk of mortality (AUC 0.85), and patients with sTTR < 20 mg/dL showed significantly reduced survival. Higher on-treatment sTTR levels at six months were associated with better functional capacity (r = 0.52; *p* = 0.009) and, when >26 mg/dL, with lower observed mortality during follow-up. **Conclusions:** In a real-world cohort of patients with ATTR-CM treated with tafamidis, serum transthyretin levels were associated with disease severity, functional status, and survival. Baseline and early on-treatment sTTR measurements may represent useful biomarkers for risk stratification and treatment monitoring.

## 1. Introduction

Transthyretin amyloid cardiomyopathy (ATTR-CM) is a progressively debilitating infiltrative cardiomyopathy caused by myocardial deposition of transthyretin (TTR) amyloid fibrils, leading to ventricular hypertrophy, diastolic dysfunction, arrhythmias, and progressive heart failure [1]. Once considered a rare condition, ATTR-CM is now increasingly recognized, largely due to the implementation of noninvasive diagnostic algorithms, and represents an important cause of heart failure in older adults [2]. ATTR-CM occurs in two main forms: a hereditary variant (ATTRv-CM), resulting from pathogenic mutations in the *TTR* gene that produce an unstable protein, and a wild-type form (ATTRwt-CM), in which amyloid deposition occurs in the absence of detectable mutations, likely driven by age-related mechanisms [1].

At the molecular level, disease pathogenesis is initiated by destabilization of the native TTR tetramer, promoting its dissociation into monomers that misfold, aggregate, and assemble into insoluble amyloid fibrils [3]. These fibrils progressively accumulate within the myocardium and other tissues, ultimately leading to structural remodeling and functional impairment.

Major advances in disease-modifying therapies have recently transformed the therapeutic landscape of ATTR-CM [4]. Currently approved treatments act either by reducing hepatic TTR synthesis through small interfering RNA (siRNA) therapies (e.g., patisiran, vutrisiran) or by stabilizing the TTR tetramer and preventing its dissociation (e.g., tafamidis, acoramidis).

Recent studies have demonstrated that pharmacological stabilization of the TTR tetramer is associated with measurable increases in circulating TTR levels, reflecting target engagement. In clinical trials, tafamidis therapy has been consistently associated with increases in sTTR concentrations [4], while newer stabilizers such as acoramidis have shown even greater elevations, consistent with their higher stabilizing potency [5]. These observations support the concept that circulating sTTR may serve as a pharmacodynamic biomarker of TTR stabilization.

In contrast, current staging systems in ATTR-CM are primarily based on biomarkers such as NT-proBNP, cardiac troponins, and estimated glomerular filtration rate, which reflect end-organ dysfunction rather than directly capturing the underlying disease pathophysiology. As a result, these markers may provide limited insight into the biological effects of disease-modifying therapies.

Despite these mechanistic insights, the clinical significance of sTTR levels in patients with ATTR-CM remains incompletely defined. In the context of expanding therapeutic options, serial assessment of sTTR may represent a potential pharmacodynamic marker reflecting the degree of TTR stabilization and, indirectly, the rate of new amyloid formation. While preliminary data from clinical trials—particularly with TTR stabilizers—suggest that sTTR may reflect target engagement, its association with disease severity, longitudinal response, and clinical outcomes in patients treated with tafamidis, especially in real-world settings, remains insufficiently defined [5,6].

We hypothesized that early increases in sTTR after initiation of tafamidis therapy might be associated with a more favorable biological and clinical profile. Accordingly, the aim of this study was to evaluate the relationship between serum TTR levels and markers of disease severity, to characterize longitudinal changes in sTTR during long-term tafamidis treatment, and to explore the association between sTTR and clinical outcomes in a real-world ATTR-CM population.

## 2. Materials and Methods

### 2.1. Study Design and Ethics

This was a retrospective, exploratory study of prospectively collected data from consecutive patients diagnosed with ATTR-CM who were evaluated between January 2022 and January 2023 at the Cardiomyopathy Center, Policlinico Umberto I University Hospital, Rome, Italy. All procedures were performed in accordance with the ethical standards of the Declaration of Helsinki. The study protocol was approved by the local institutional ethics committee, and written informed consent was obtained from all participants.

### 2.2. Patient Population

Patients were eligible for inclusion if they had a confirmed diagnosis of ATTR-CM based on clinical presentation and imaging criteria in accordance with current ESC guidelines [7]. Specifically, ATTR-CM was diagnosed in the presence of echocardiographic and/or cardiac magnetic resonance (CMR) findings suggestive of cardiac amyloidosis and further confirmed by technetium-99m (99mTc)-labeled bone scintigraphy showing a Perugini score > 1, in the absence of monoclonal proteins on serum and urine immunofixation electrophoresis. In cases of equivocal scintigraphic findings (Perugini score = 1) or the presence of monoclonal gammopathy, the diagnosis was definitively established by histology. Peripheral tissue biopsy (periumbilical fat pad or salivary gland) was initially performed, followed by endomyocardial biopsy when the peripheral tissue was negative. Amyloid deposits were identified by Congo red staining, and fibril type was determined using immunohistochemical analysis with AmYkit (Amimed) antibodies. All patients were tafamidis-naïve at the time of enrollment and initiated therapy at the approved dose of 61 mg once daily. None of the patients had received prior treatment with *TTR* gene silencing therapies (including inotersen, patisiran, or vutrisiran), nor were such therapies initiated during the study period.

Exclusion criteria included the presence of active systemic inflammatory or autoimmune diseases, severe hepatic dysfunction, nephrotic syndrome or other protein-losing conditions, clinically overt malnutrition, and any other condition considered by the investigators to potentially interfere with a reliable interpretation of serum TTR measurements.

### 2.3. Laboratory Assessment

sTTR concentrations were measured at baseline and after six months of tafamidis therapy, based on prior evidence showing an early and stable increase in sTTR following initiation of TTR stabilizer treatment. All laboratory analyses were performed at a single accredited central laboratory. Serum TTR levels were quantified using a photometric assay on a Roche Diagnostics Cobas analyzer (Roche Diagnostics GmbH, Mannheim, Germany, reference range: 15–40 mg/dL). To account for potential confounding related to nutritional status, serum albumin levels were measured concurrently. Body surface area (BSA) was calculated at both time points to further adjust for physiological variability.

### 2.4. Clinical Assessment and Follow-Up

Patients underwent a comprehensive baseline clinical evaluation, including functional status, laboratory testing, electrocardiography, and imaging assessment. Routine follow-up visits were scheduled approximately every six months according to standard clinical practice. Patients were followed for a minimum of 24 months. Clinical events were collected during scheduled follow-up visits every six months. For patients who did not attend in-person visits, structured telephone interviews were performed to ascertain survival status and the occurrence of clinical events. No patients were lost to follow-up for survival status.

### 2.5. Study Endpoints

The primary endpoint was the change in serum transthyretin (sTTR) levels from baseline to six months after initiation of tafamidis therapy and their association with all-cause mortality.

Secondary endpoints included the association between baseline sTTR levels and markers of disease severity, including functional capacity (six-minute walk test, 6MWT); patient-reported health status (Kansas City Cardiomyopathy Questionnaire, KCCQ); Mayo disease stage; and biochemical markers (NT-proBNP and cardiac troponin T-hs).

The clinical and prognostic relevance of on-treatment sTTR levels at six months during tafamidis therapy, including the evaluation of a prognostically relevant sTTR cut-off derived from ROC analysis, and their association with survival, as well as with 6MWT, KCCQ, Mayo disease stage, and biochemical markers (NT-proBNP and cardiac troponin T-hs).

### 2.6. Statistical Analysis

Continuous variables are presented as mean ± standard deviation or median [interquartile range], as appropriate, and categorical variables are expressed as counts (percentages). Normality of data distribution was assessed using the Shapiro–Wilk test. Group comparisons were performed using the Mann–Whitney U test or Student’s *t*-test for continuous variables and the χ^2^ test or Fisher’s exact test for categorical variables, as appropriate. Associations between sTTR levels and clinical or biochemical parameters were assessed using Pearson or Spearman correlation coefficients, according to data distribution. Cox proportional hazards regression was used to evaluate predictors of all-cause mortality. A multivariable Cox model including sTTR levels and age was constructed; given the limited number of outcome events, multivariable analyses were restricted to a small set of clinically relevant variables to minimize the risk of model overfitting. ROC curve analysis, using the Youden index, was performed to identify optimal baseline and on-treatment (six-month) sTTR cut-off values for mortality prediction. Survival analyses were conducted using Kaplan–Meier curves and compared using the log-rank test. Missing data were handled using a complete-case approach, with each analysis including only patients with available data for the variables of interest. No imputation procedures were performed. Survival analyses were based on complete follow-up information for all enrolled patients. A two-sided *p*-value < 0.05 was considered statistically significant. All statistical analyses were performed using SPSS software (version 28.0.0; IBM Corp., Armonk, NY, USA).

## 3. Results

Between January 2022 and January 2023, 107 consecutive patients with ATTR-CM treated with tafamidis (93 ATTR wild-type, 14 ATTR variant; 84 males; mean age 79.6 ± 8.5 years) were prospectively enrolled and followed for a mean period of 24 ± 6 months. Baseline characteristics of the study population are reported in Table 1, and longitudinal follow-up data are summarized in Supplementary Table S1. Except for age, no statistically significant differences were observed between patients with ATTRv-CM and ATTRwt-CM. Serum TTR levels in the overall population were 24.7 ± 9.0 (normal value 15–40 mg/dL). Notably, ATTRv patients showed a non-significant trend toward higher baseline sTTR levels compared with ATTRwt patients, possibly reflecting the older age and more advanced clinical profile of the ATTRwt group in our cohort.

### 3.1. Change in Serum TTR on Tafamidis Therapy

**Figure 1:**
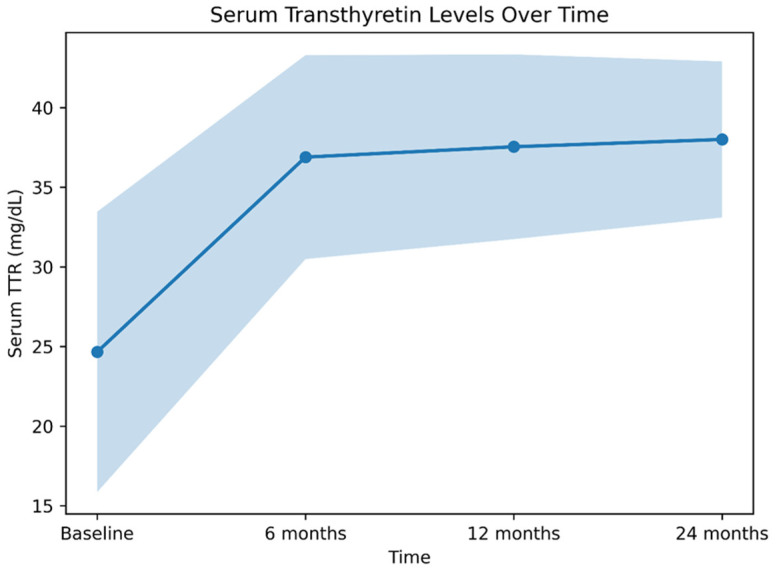
sTTR levels over time during tafamidis therapy. Mean sTTR increased from 24.66 ± 8.8 mg/dL at baseline to 36.89 ± 6.4 mg/dL at 6 months (+49.56%) and remained stable at 12 months (37.54 ± 5.8 mg/dL) and 24 months (38.0 ± 4.9 mg/dL). The shaded area indicates standard deviation.

In the overall population, tafamidis therapy was associated with a significant increase in serum TTR (sTTR) levels at 6 months, with a mean relative increase of approximately 50% (from 24.66 ± 8.8 to 36.89 ± 6.4 mg/dL; *p* = 0.0061), corresponding to a mean absolute increase of 12.2 mg/dL. sTTR levels at 12 and 24 months showed a further slight increase compared with 6 months (37.54 ± 5.8 mg/dL and 38.0 ± 4.9 mg/dL, respectively), which did not reach statistical significance (*p* = 0.41 and *p* = 0.39 vs. 6 months, respectively) (Figure 1). In contrast, no significant changes were observed in serum albumin levels (3.75 ± 0.42 g/dL at baseline vs. 3.78 ± 0.38 g/dL at 6 months; *p* = 0.518) or body surface area (BSA) (1.74 ± 0.16 m^2^ at baseline vs. 1.73 ± 0.15 m^2^ at 6 months; *p* = 0.632), suggesting that the observed increase in sTTR was unlikely to be driven by changes in nutritional status.

### 3.2. Baseline sTTR and Disease Severity

**Figure 2:**
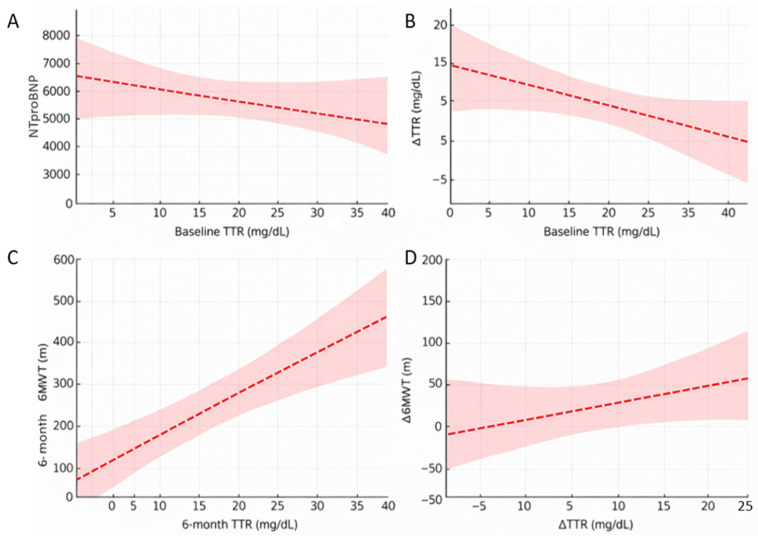
Correlations between baseline TTR and NT-proBNP (**A**), baseline TTR and ∆TTR at 6 months (**B**), 6-month TTR and change in 6 min walk test distance at 6 months (**C**), and ∆TTR and 6MWT at 6 months (**D**). The dashed line represents the fitted linear regression model.

Baseline sTTR levels showed a significant inverse correlation with NT-proBNP (ρ = −0.349; *p* = 0.037) (Figure 2A). No significant associations were observed between baseline sTTR levels and Mayo disease stage, cardiac troponin levels, or KCCQ scores. Baseline sTTR levels were also inversely correlated with the magnitude of tafamidis-induced sTTR increase at six months (ρ = −0.72; *p* < 0.001) (Figure 2B). These findings suggest that lower baseline sTTR levels are associated with a more advanced heart failure profile.

### 3.3. sTTR Changes and Clinical Response

Higher on-treatment sTTR levels at six months were positively associated with functional capacity, as assessed by the six-minute walk test (6MWT) distance (r = 0.52; *p* = 0.009) (Figure 2C). Similarly, changes in sTTR levels from baseline to six months correlated with changes in 6MWT over the same period (r = 0.49; *p* = 0.017) (Figure 2D). No significant correlations were observed between sTTR variations and changes in NT-proBNP, cardiac troponin levels, or KCCQ scores. Collectively, these results indicate that higher on-treatment sTTR levels and greater sTTR increases are associated with better functional status and functional improvement.

### 3.4. Prognostic Significance of sTTR

During follow-up, 12 patients died. Comparisons between survivors and non-survivors are reported in Table 2. Non-survivors had significantly lower baseline sTTR levels compared with survivors (14.2 ± 3.1 vs. 23.5 ± 5.2 mg/dL; *p* < 0.001). In addition, non-survivors exhibited higher baseline high-sensitivity troponin levels (82.0 [56.2–129.9] vs. 57.1 [25.0–103.0] pg/mL; *p* = 0.048), higher NT-proBNP concentrations (2832 [2826–4717] vs. 1404 [561–3702] pg/mL; *p* = 0.020), and lower right ventricular systolic function, as reflected by reduced TAPSE values (18.0 [16.0–18.0] vs. 20.0 [18.0–23.0] mm; *p* = 0.022). Age did not differ significantly between survivors and non-survivors.

**Figure 3:**
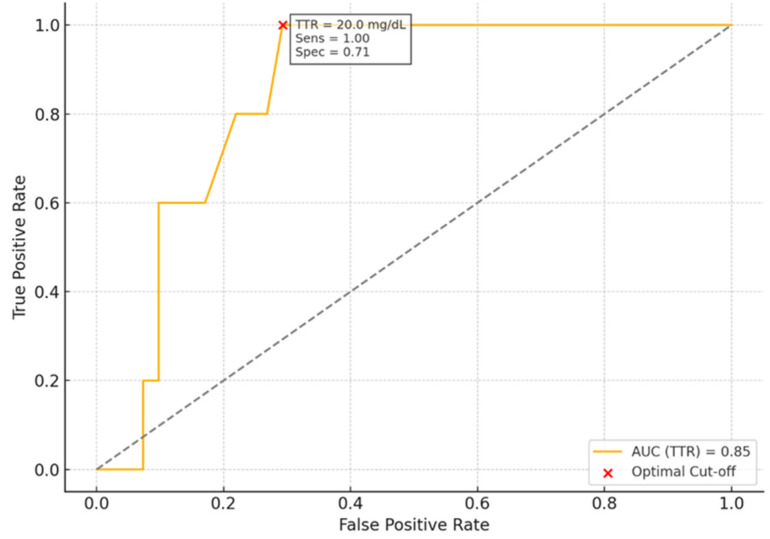
ROC curve showing the predictive performance of baseline TTR levels for mortality. The optimal cut-off point (TTR = 20.0 mg/dL) is highlighted in red, corresponding to a sensitivity of 100% and a specificity of 71%, as determined by the Youden index.

**Figure 4:**
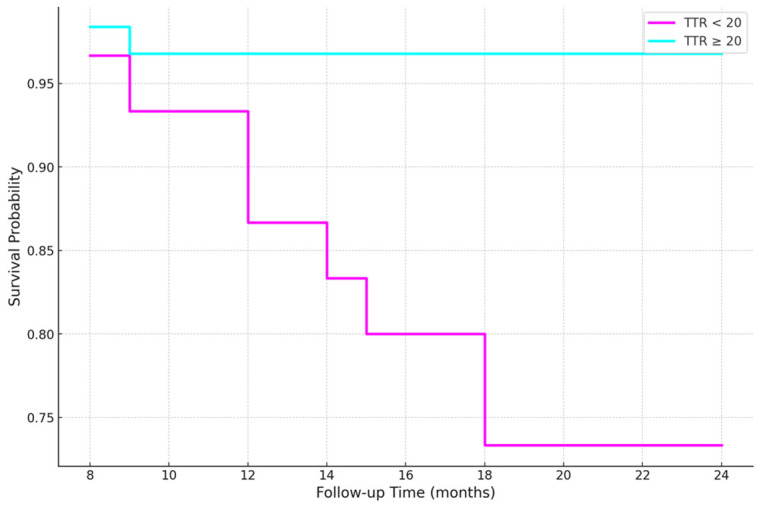
Kaplan–Meier survival curves stratified by baseline sTTR levels. Patients with TTR < 20 mg/dL showed reduced survival compared to those with TTR ≥ 20 mg/dL over the follow-up period (log-rank *p* = 0.00076).

In Cox regression analysis, each 1 mg/dL increase in baseline sTTR was associated with a 13% reduction in all-cause mortality risk (HR 0.87, 95% CI 0.83–0.91; *p* < 0.001) (Table 3). ROC analysis identified 20 mg/dL as the optimal baseline sTTR cut-off for mortality prediction (AUC 0.85; sensitivity 100%, specificity 71%) (Figure 3). Patients with baseline sTTR < 20 mg/dL had a markedly higher risk of death during follow-up (HR 9.2, 95% CI 1.96–43.4; *p* = 0.005). Kaplan–Meier survival curves stratified by baseline sTTR levels showed significantly reduced survival among patients with sTTR < 20 mg/dL compared with those with sTTR ≥ 20 mg/dL over the follow-up period (log-rank *p* = 0.00076) (Figure 4).

In the multivariable Cox model including age, baseline sTTR < 20 mg/dL remained independently associated with mortality (HR 9.25, 95% CI 1.96–43.7; *p* = 0.005), whereas age was not significantly associated with outcomes (Table 3).

Finally, post-treatment sTTR levels > 26 mg/dL measured at six months were associated with lower observed all-cause mortality over the 24-month follow-up (0% vs. 25% mortality; AUC 0.83).

## 4. Discussion

Transthyretin amyloid cardiomyopathy (ATTR-CM) is increasingly recognized as a prevalent cause of heart failure in older adults and is no longer considered a rare disease [2]. Parallel to advances in noninvasive diagnostic pathways, the therapeutic landscape of ATTR-CM has rapidly expanded over the last decade, with several disease-modifying therapies approved and additional agents currently under clinical investigation [4]. In this evolving scenario, there is a growing need to identify biomarkers capable of refining baseline risk stratification and, importantly, providing early insight into biological and clinical response to therapy.

### 4.1. Baseline sTTR as a Marker of Disease Severity and Prognosis

Current staging and prognostic models for ATTR-CM rely primarily on biomarkers reflecting end-organ dysfunction, such as estimated glomerular filtration rate, NT-proBNP, and cardiac troponins [8,9]. Although these parameters are powerful predictors of outcome, they largely capture downstream consequences of myocardial amyloid infiltration and heart failure rather than directly reflecting disease pathogenesis or target engagement. Moreover, these biomarkers are substantially influenced by concomitant heart failure therapies—including diuretics, mineralocorticoid receptor antagonists, and sodium–glucose cotransporter-2 inhibitors—which may limit their reliability for assessing response to disease-modifying treatments such as tafamidis [10].

From this perspective, circulating serum transthyretin (sTTR), which lies at the core of ATTR amyloid formation and represents the direct pharmacological target of stabilizing therapies, emerges as an attractive candidate biomarker. However, evidence supporting its clinical utility remains limited and is largely derived from post hoc or exploratory analyses of clinical trials evaluating novel TTR stabilizers, with virtually no real-world data available in patients treated with tafamidis [10]. To date, real-world evidence on sTTR dynamics during tafamidis therapy remains scarce. A prior real-world study demonstrated that tafamidis treatment is associated with an increase in circulating sTTR levels, supporting its pharmacodynamic effect, but did not provide insights into the clinical significance of this increase [10]. More recently, a post hoc analysis of a randomized clinical trial with acoramidis showed that early increases in sTTR are associated with improved survival [11]. However, these findings were derived from a controlled trial setting and with a different TTR stabilizer, potentially limiting their generalizability to routine clinical practice and to patients treated with tafamidis.

In this context, the present real-world study provides several novel observations. First, baseline sTTR levels were inversely correlated with NT-proBNP, supporting the concept that lower circulating TTR may reflect greater tetramer instability and, consequently, a higher burden of myocardial amyloid deposition and heart failure severity. Notably, the relatively wide distribution of baseline sTTR values observed in our cohort likely reflects the heterogeneity of a real-world ATTR-CM population, encompassing patients across different disease stages and clinical conditions.

In addition, baseline sTTR levels were significantly lower among patients who subsequently died and were strongly associated with prognosis, with each 1 mg/dL increase associated with a meaningful reduction in mortality risk.

A baseline cut-off of 20 mg/dL may help identify patients at markedly higher risk of death. Taken together, these findings suggest that sTTR may complement existing staging systems by providing additional prognostic information more closely linked to disease biology rather than downstream organ dysfunction.

### 4.2. On-Treatment sTTR Dynamics and Response to Tafamidis

The current therapeutic armamentarium for ATTR-CM includes multiple drug classes, such as TTR stabilizers, TTR protein synthesis suppressors, and TTR amyloid depleters, highlighting the need for class-specific biomarkers of treatment response. TTR stabilizers exert their therapeutic effect by binding to and stabilizing the native circulating TTR tetramer, thereby slowing its dissociation into monomers that can generate toxic amyloidogenic species. In vivo, effective TTR stabilization may be reflected by increased circulating sTTR levels [11].

Although TTR stabilizers as a drug class are generally associated with rises in sTTR, the magnitude of this increase varies across agents and appears closely related to the degree of tetramer stabilization achieved. This biological framework provides a strong rationale for investigating sTTR as a pharmacodynamic marker specifically relevant to TTR stabilizer therapy.

In our study, tafamidis therapy was associated with a substantial and early increase in circulating sTTR levels, which remained stable over longer-term follow-up. This temporal pattern is consistent with the known mechanism of action of tafamidis, which stabilizes the TTR tetramer and increases circulating intact TTR. Notably, the magnitude of sTTR increase was inversely related to baseline sTTR values, suggesting a larger biochemical response in patients with lower starting levels.

This observation may reflect greater availability of unstable tetramers at baseline or a higher degree of pharmacological stabilization in more advanced disease stages, although mechanistic inferences should be interpreted cautiously. Importantly, patients achieving on-treatment sTTR levels > 26 mg/dL showed a more favorable observed survival profile, supporting the potential prognostic relevance of treatment-induced sTTR changes.

### 4.3. Relationship Between sTTR and Functional Outcomes

Higher on-treatment sTTR levels and greater increases in sTTR were associated with better functional status and functional improvement, as assessed by six-minute walk distance. Functional capacity, health-related quality of life, and survival represent key clinical endpoints in pivotal trials of disease-modifying therapies for ATTR-CM, including tafamidis [5,6], underscoring the clinical relevance of these associations.

In contrast, changes in sTTR were not associated with short-term variations in NT-proBNP, cardiac troponin, or KCCQ scores. This lack of association may, at least in part, be explained by the substantial influence of concomitant heart failure therapies commonly initiated or optimized during follow-up, which may limit the ability of these markers to capture disease-modifying effects. The observed relationship between higher on-treatment sTTR levels and improvement in six-minute walk distance does not imply causality but supports the potential utility of sTTR as a clinically meaningful biomarker reflecting biological response in parallel with functional status.

### 4.4. Clinical Implications and Future Perspectives

Taken together, these findings suggest a potential dual role for sTTR in ATTR-CM: as a baseline marker of disease severity and prognosis and as an early pharmacodynamic indicator associated with functional response to TTR stabilizer therapy. With further validation in larger studies, sTTR could be integrated into existing disease staging systems to improve risk stratification and potentially inform therapeutic decision-making. Such a biomarker may become increasingly valuable in an era of multiple therapeutic options, where early identification of patients unlikely to derive meaningful benefit from a given therapy—or who may require alternative or combination strategies—will be of growing clinical importance.

## 5. Limitation

This was a single-center, exploratory study with a relatively small sample size and a limited number of outcome events, which may reduce statistical power and limit the generalizability of the findings. Consequently, multivariable analyses were necessarily restricted, and residual confounding cannot be excluded. In addition, we did not adjust for concomitant heart failure therapies, which may have influenced both biomarker levels and clinical outcomes. Although serum albumin was measured and patients with conditions known to markedly affect circulating TTR levels were excluded, sTTR concentrations may still be influenced by factors beyond ATTR pathobiology, including nutritional status and intercurrent illness.

The observational design precludes causal inference, and the sTTR cut-off values identified in this study should be considered hypothesis-generating rather than definitive.

Finally, all patients were treated with tafamidis; therefore, the applicability of these findings to other TTR-stabilizing agents or therapies acting through different mechanisms cannot be assumed.

## 6. Conclusions

In a real-world cohort of patients with transthyretin amyloid cardiomyopathy treated with tafamidis, serum transthyretin levels were associated with disease severity, functional status, and survival. Lower baseline sTTR levels were associated with a higher risk of mortality, while higher on-treatment sTTR levels at six months were associated with more favorable outcomes.

These findings support the potential role of sTTR as an easily accessible biomarker for baseline risk stratification and treatment monitoring in ATTR-CM. Larger, multicenter prospective studies are warranted to validate these observations and to define the clinical utility of sTTR within integrated prognostic and therapeutic algorithms.

## Figures and Tables

**Table 1:** Baseline characteristics of the study population according to genotype.

Variable	Overall (107)	ATTRwt (93)	ATTRv (14)	*p*-Value
Age, years	83.00 (72.00–87.00)	83.00 (74.00–87.00)	70.0 (60.0–71.0)	0.001
Serum TTR, mg/dL	24.7 ± 9.0	24.3 ± 9.3	27.72 ± 6.30	0.318
KCCQ score	83.22 ± 23.16	82.41 ± 23.34	89.80 ± 21.64	0.343
6MWT, m	405.00 (311.75–441.75)	405.00 (305.25–432.00)	459.00 (432.00–486.00)	0.059
Troponin T-hs ng/L	57.70 (26.00–106.40)	59.00 (28.70–106.40)	24.20 (5.76–39.60)	0.013
NT-proBNP, pg/mL	1578.00 (603.00–4266.00)	1716.00 (603.00–4266.00)	1177.00 (735.00–1558.00)	0.283
eGFR, mL/min/1.73 m^2^	56.80 (44.48–77.33)	55.50 (43.00–77.10)	68.72 ± 13.59	0.087
Gillmore stage	2 (2–3)	2 (2–3)	2 (2–2)	0.158
MAYO stage	3 (3–3)	3 (3–3)	3 (3–3)	0.370
LVEF, %	50.00 (45.00–55.00)	50.00 (45.00–55.00)	55.00 (45.00–55.00)	0.945
TAPSE, mm	19.00 (17.00–23.00)	19.00 (18.00–23.00)	19.00 (15.00–25.00)	0.885
IVS thickness, mm	18.34 ± 3.14	18.36 ± 3.22	17.00 (16.00–20.00)	0.848
Atrial fibrillation	42 (44.7%)	38 (45.2%)	4 (40.0%)	1.000
Hypertension	76 (80.9%)	70 (83.3%)	6 (60.0%)	0.094
Diabetes mellitus	20 (21.3%)	20 (23.8%)	0 (0.0%)	0.113
Coronary artery disease	22 (23.4%)	22 (26.2%)	0 (0.0%)	0.110
Prior TIA/Stroke	8 (8.5%)	8 (9.5%)	0 (0.0%)	0.593
Pacemaker	16 (17.8%)	16 (20.0%)	0 (0.0%)	0.199
ICD	4 (4.3%)	2 (2.4%)	2 (20.0%)	0.055
CRT-P	2 (2.1%)	2 (2.4%)	0 (0.0%)	1.000
CRT-D	2 (2.1%)	2 (2.4%)	0 (0.0%)	1.000

Continuous variables are reported as mean ± standard deviation (SD) when normally distributed and as median (interquartile range, IQR) when non-normally distributed. Categorical variables are expressed as numbers (prevalence, %). *p*-values refer to comparisons between ATTRwt and ATTRv groups.

**Table 2:** Baseline characteristics of the study population; comparison of clinical and biochemical characteristics between non-survivors and survivors.

Variable	Overall (107)	Survivors (95)	Non-Survivors (12)	*p*-Value
Age, years	83.00 (72.00–87.00)	83.00 (72.00–87.00)	80.00 ± 5.89	0.830
Serum TTR, mg/dL	24.00 (17.00–33.20)	23.5 ± 5.2	14.2 ± 3.1	0.001
KCCQ score	83.22 ± 23.16	83.12 ± 23.64	84.00 ± 19.91	0.985
6MWT, m	405.00 (311.75–441.75)	411.50 (322.25–441.75)	354.50 ± 155.81	0.485
Troponin T-hs, ng/L	57.70 (26.00–106.40)	57.10 (25.00–103.00)	82.00 (56.20–129.90)	0.048
NT-proBNP, pg/mL	1578.00 (603.00–4266.00)	1404.00 (561.00–3701.90)	2832 (2826–4717)	0.020
eGFR, mL/min/1.73 m^2^	56.80 (44.48–77.33)	59.65 (45.90–77.40)	52.00 (32.00–55.00)	0.179
Gillmore stage	2 (2–3)	2 (2–3)	3 (2–3)	0.197
MAYO stage	3 (3–3)	3 (3–3)	3 (3–3)	0.236
LVEF, %	50.00 (45.00–55.00)	51.00 (45.00–55.00)	46.00 (45.00–55.00)	0.222
TAPSE, mm	19.00 (17.00–23.00)	20.00 (18.00–23.00)	17.20 ± 1.55	0.022
IVS thickness, mm	18.34 ± 3.14	18.21 ± 3.11	19.40 ± 3.37	0.219
ATTRv (mutation)	10 (10.6%)	10 (11.9%)	0 (0.0%)	0.593
Atrial fibrillation	42 (44.7%)	36 (42.9%)	6 (60.0%)	0.334
Hypertension	76 (80.9%)	70 (83.3%)	6 (60.0%)	0.094
Diabetes mellitus	20 (21.3%)	20 (23.8%)	0 (0.0%)	0.113
Coronary artery disease	22 (23.4%)	22 (26.2%)	0 (0.0%)	0.110
Prior TIA/Stroke	8 (8.5%)	6 (7.1%)	2 (20.0%)	0.201
Pacemaker	16 (17.8%)	14 (16.7%)	2 (33.3%)	0.288
ICD	4 (4.3%)	4 (4.8%)	0 (0.0%)	1.000
CRT-P	2 (2.1%)	2 (2.4%)	0 (0.0%)	1.000
CRT-D	2 (2.1%)	0 (0.0%)	2 (20.0%)	0.010

Data presented as mean/prevalence ± standard deviation or median (IQR).

**Table 3:** Univariable Cox proportional hazards regression analysis for all-cause mortality at 24 months.

Variable	Hazard Ratio (HR)	95% Confidence Interval	*p* Value
sTTR (per 1 mg/dL increase)	0.87	0.83–0.91	<0.001
Age (per 1-year increase)	1.01	0.94–1.08	0.876
NT-proBNP, baseline (log10)	5.74	1.15–28.59	0.033
Troponin baseline (log10)	12.15	1.56–94.46	0.017
EF (per 1% increase)	0.94	0.86–1.03	0.163
IVS (per 1 mm increase)	1.12	0.92–1.37	0.269
TAPSE, baseline (per 1 mm increase)	0.80	0.65–0.98	0.031
Gilmore score, baseline (per 1-point increase)	2.39	0.67–8.48	0.177
eGFR, baseline (per 1 mL/min/1.73 m^2^ increase)	0.98	0.95–1.01	0.233
Hypertension (yes vs. no)	0.34	0.10–1.20	0.094
History of TIA or stroke (yes vs. no)	2.56	0.54–12.04	0.235
Atrial fibrillation (yes vs. no)	1.94	0.55–6.88	0.304
6MWT (per 1 m increase)	1.00	0.99–1.00	0.364
KCCQ (per 1-point increase)	1.00	0.97–1.03	0.961

## Data Availability

The data supporting the findings of this study are available from the corresponding author upon reasonable request and in accordance with applicable data protection regulations.

## Supplementary Materials

The following supporting information can be downloaded at: https://www.mdpi.com/article/10.3390/jcm15093355/s1, Table S1. longitudinal changes during follow-up.

## Abbreviations

The following abbreviations are used in this manuscript:

ATTR-CM	Transthyretin amyloid cardiomyopathy
ATTRv-CM	Hereditary transthyretin amyloid cardiomyopathy
ATTRwt-CM	Wild-type transthyretin amyloid cardiomyopathy
TTR	Transthyretin
sTTR	Serum transthyretin
CMR	Cardiac magnetic resonance
ESC	European Society of Cardiology
99mTc	Technetium-99m
BSA	Body surface area
6MWT	Six-minute walk test
KCCQ	Kansas City Cardiomyopathy Questionnaire
ROC	Receiver operating characteristic
AUC	Area under the curve
IQR	Interquartile range
TAPSE	Tricuspid annular plane systolic excursion
siRNA	Small interfering RNA
