# Supplementary material for: Serum Transthyretin as a Prognostic and Therapeutic Biomarker in Transthyretin Amyloid Cardiomyopathy Patients Treated with Tafamidis
Source: J Clin Med. 2026 Apr 28;15(9):3355. doi: 10.3390/jcm15093355 (PMC13164462; doi:10.3390/jcm15093355)
Supplement: Supplementary file 1 [file jcm-15-03355-s001.zip › jcm-4189372-supplementary.pdf]

Supplementary Table S1. longitudinal changes during follow-up

Variable	Baseline	6 months	12 months	24 months
**Serum TTR, mg/dL**	24.7 ± 9.0	36.9 ± 6.4	37.2 ± 6.0	37.0 ± 5.8
**KCCQ score**	83.2 ± 23.2	85.5 ± 22.0	81.0 ± 22.5	75.2 ± 23.0
**6MWT, m**	405 (311.8–441.8)	418 (330–450)	392 (310–430)	385 (285–405)
**Troponin T hs ng/L**	57.7 (26.0–106.4)	55.0 (25.0–101.0)	58.0 (27.0–108.0)	62.0 (30.0–115.0)
**NT-proBNP, pg/mL**	1578 (603–4266)	1480 (580–3950)	1620 (620–4200)	1780 (700–4550)
**eGFR, mL/min/1.73 m²**	56.8 (44.5–77.3)	55.8 (43.8–76.0)	50.8 (42.0–74.0)	45.5 (40.0–71.0)
**Gillmore stage**	2 (2–3)	2 (2–3)	2 (2–3)	2 (2–3)
**MAYO stage**	3 (3–3)	3 (3–3)	3 (3–3)	3 (3–3)
**LVEF, %**	50 (45–55)	50 (45–55)	49 (44–54)	48 (43–53)
**TAPSE, mm**	19 (17–23)	19 (17–23)	18 (16–22)	18 (16–21)
**IVS thickness, mm**	18.3 ± 3.1	18.4 ± 3.1	18.6 ± 3.2	18.9 ± 3.2

Supplementary Table S1 summarizes longitudinal changes in clinical, laboratory, functional, and echocardiographic parameters during follow-up at 6, 12, and 24 months after tafamidis initiation. Values are expressed as mean ± standard deviation or median (interquartile range), as appropriate.
